# Comparison of cortisol and inflammatory response between aged and middle-aged patients undergoing total hip arthroplasty: a prospective observational study

**DOI:** 10.1186/s12891-017-1900-y

**Published:** 2017-12-19

**Authors:** Jian Zhong, Hai-bo Si, Yi Zeng, Jing Yang, Zong-ke Zhou, Peng-de Kang, Fu-xing Pei, Bin Shen

**Affiliations:** Department of orthopaedics, West China Hospital, Sichuan University, Chengdu, 610041 People’s Republic of China

**Keywords:** Arthroplasty, Hip, Cortisol, Aged, Middle aged

## Abstract

**Background:**

To investigate the differences in the perioperative serum cortisol, C-reactive protein (CRP) and interleukin-6 (IL-6) levels between aged and middle-aged patients undergoing total hip arthroplasty (THA).

**Methods:**

Sixty patients (30 aged and 30 middle-aged) undergoing THA for osteoarthritis between August 2016 and January 2017 participated in this study. Blood samples were collected preoperatively and at 6 hours, 24 hours and 3 days after surgery to measure the cortisol, CRP and IL-6 concentrations. The clinical outcomes were assessed using the visual analogue scale (VAS) pain score and Harris hip score (HHS).

**Results:**

No significant differences were found between the two groups before the operation in the cortisol, IL-6 and CRP levels; the VAS score; or the HHS. Cortisol was significantly lower at 6 hours after surgery in the aged group than in the middle-aged group (*P* < 0.05). IL-6 at 6 and 24 hours after surgery, CRP at 3 days after surgery and the VAS score at 6 and 24 hours after surgery in the aged group were significantly higher than those in the middle-aged group (*P* < 0.05). In the aged group, weak correlations were found between the cortisol concentration 6 hours after THA and the IL-6 level 24 hours after THA (r = −0.37, *P* = 0.04) and between the IL-6 level 6 hours after THA and the VAS score 24 hours after THA (r = 0.42, *P* = 0.02).

**Conclusion:**

Aged patients showed lower cortisol levels at 6 hours after surgery and higher IL-6 levels at 6 and 24 hours after surgery than middle-aged patients undergoing THA.

## Background

Life expectancy has been prolonged considerably due to improved healthcare. The prevalence of osteoarthritis (OA) is almost 70% in women and 60% in men over the age of 65 years, and most people who suffer from OA display radiographic evidence and/or clinical symptoms [1, 2]. Total hip arthroplasty (THA) is an effective method to ease pain and improve the quality of life for people who suffer from serious pain and disability caused by OA [3–6].

However, surgeries can trigger stress responses, including a series of immunological, metabolic and hormonal changes [7–10]. As one crucial constituent of the stress response, cortisol secretion is inevitably increased after surgery [11, 12]. Human ageing causes homeostasis disturbances in all systems, with a progressive loss of structural and physiological integrity and function [13]. In particular, impairment of the suprachiasmatic nucleus, which affects the limbic region, hippocampus and hypothalamus, leads to adrenocortical secretion management disorders in aged people [14, 15].

Although several studies have compared cortisol levels between aged and middle-aged patients undergoing other operations, the conclusions have been inconsistent [16–19]. To the best of our knowledge, no study in the major English language medical databases has reported the cortisol levels of aged and middle-aged patients undergoing THA. We performed this study to investigate differences in cortisol levels between aged and middle-aged patients undergoing THA and the relationship between endogenous cortisol and the inflammatory response after the operation. We hypothesized that aged patients undergoing THA would show a higher level of cortisol and lower interleukin-6 (IL-6) and C-reactive protein (CRP) levels postoperatively than middle-aged patients due to the anti-inflammatory effect of endogenous cortisol production.

## Method

### Patients

Patients aged over 40 years and scheduled for primary unilateral uncemented THA for OA between August 2016 and January 2017 were screened in this prospective, non-randomized, observational study. The exclusion criteria were as follows: senile dementia, steroid use within the past 6 months, chronic malnutrition, smoking, alcoholism, drug abuse, rheumatoid arthritis, hypertension, diabetes mellitus and other systemic diseases that would influence the neuroendocrine and inflammatory responses. The administration of non-steroidal anti-inflammatory drugs (NSAIDs) was suspended four weeks before the operation. Of the 68 eligible patients, 7 patients refused to participate, and 1 patient withdrew from this study. Finally, 60 patients were included in the study. The patients were divided into an aged group (over 65 years) and a middle-aged group (40–65 years). This study was conducted in accordance with the Declaration of Helsinki and approved by the local Ethics Committee. All patients gave informed consent prior to their inclusion in the study.

### Surgical procedure

All surgeries were performed by one single experienced surgeon (B.S.). Generally, 1.5 g of cefuroxime was given within 30 min before the beginning of surgery, and general anaesthesia was used in all cases. The same operative technique was used. Through the posterolateral approach, the acetabular component was placed at 20° anteversion and 45° abduction, the femoral component was fixed at 15° anteversion, the capsular flap and the short external rotators were repaired in all cases, and wound drainage was indwelled in every patient.

### Perioperative management

Preadmission counselling was given by qualified nurses, and the preliminary assessment was conducted by a doctor. Clear oral fluids were administered up to 3 h before the operation to minimize preoperative fasting. No sedative was used before surgery. One hundred milligrams of ropivacaine (100 mg: 10 ml) was diluted with 30 ml of saline and subcutaneously injected around the wound as a local infiltration analgesia.

Forty milligrams of parecoxib was given by intramuscular injection at 6 h after surgery. A second dose of 40 mg of parecoxib was given at 8:00 a.m. on postoperative day (POD) 1, followed by oral celecoxib (200 mg) every 12 h. Ten milligrams of rivaroxaban was given orally 8 h after the operation and then administered at 24-h intervals on the subsequent days provided that no bleeding events occurred.

At 8:00 a.m. on POD 1, wound drainage and the monitor were removed, and unrestricted food intake was permitted. Daily functional training, including walking and muscle power exercises, was guided and supervised by a physiotherapist.

### Outcome assessments

Venous blood samples were collected in the morning of the day before surgery from each patient as baseline. The primary outcome was the serum cortisol level. Blood samples to measure the cortisol, CRP and IL-6 levels were obtained at 6 h, 24 h and 3 days postoperatively. After removing cells and debris by centrifugation, the samples were stored at −80 °C prior to the analysis. All measurements were performed at the clinical key laboratory of West China Hospital, which was certificated by the College of American Pathologists (CAP). CRP was measured using the rate nephelometric assay on the IMMAGE®800 Access immunoassay system (Beckman Coulter Inc., Brea, CA, USA) and reported in milligrams per litre (mg/L). IL-6 and cortisol were measured with electrochemiluminescence immunoassays (ECLIs) on the modular analytics E170 module (Roche Diagnostics, Mannheim, Germany) and were presented in picograms per millilitre (pg/mL) and nanomole per litre (nmol/L), respectively.

Visual analogue scale (VAS) pain scores (rest pain) were marked on the day of hospitalization and 6 h, 24 h, 2 days, and 3 days after surgery by a mediatinus (no pain = 0, worst pain = 10). The Harris hip scores (HHS) were obtained at the time of hospital admission and 3 months postoperatively to evaluate the early recovery effect. Adverse events were recorded at each follow-up.

### Statistical analysis

Data were collected using Excel. Continuous variables are presented as the means and standard deviations (M ± SD). Dichotomous data are presented as numbers for each category. To compare data between two groups, the normality of all quantitative data was tested with the Shapiro-Wilk test. For normally distributed data, Levene’s test for equality of variances was used to test the homogeneity of variances of matching data from two groups, and the two independent samples *t*-test was adopted if the variances were homogenous; otherwise, a *t*-test was performed. For non-normally distributed and ranked data, a nonparametric test (Mann-Whitney rank-sum test) was conducted. For enumeration data, the Chi-square test was employed. Because some of our variables (cortisol before and 24 h after surgery in the middle-aged group, IL-6 at 6, 24 h and 3 days after surgery in the middle-aged group, and CRP before and at 6 h after surgery in both groups) were not normally distributed, analysis of variance (ANOVA) of repeated measurement data was not used. Pearson’s correlation coefficient and Spearman’s rank correlation coefficient were calculated for variables with normal and non-normal distributions, respectively, within each group to analyse their possible correlations. The analyses were performed using the SPSS statistical software (Version 13.0, SPSS Inc., Chicago, IL, USA). For all comparisons, a *P* value <0.05 was considered significant.

## Results

**Fig. 1:**
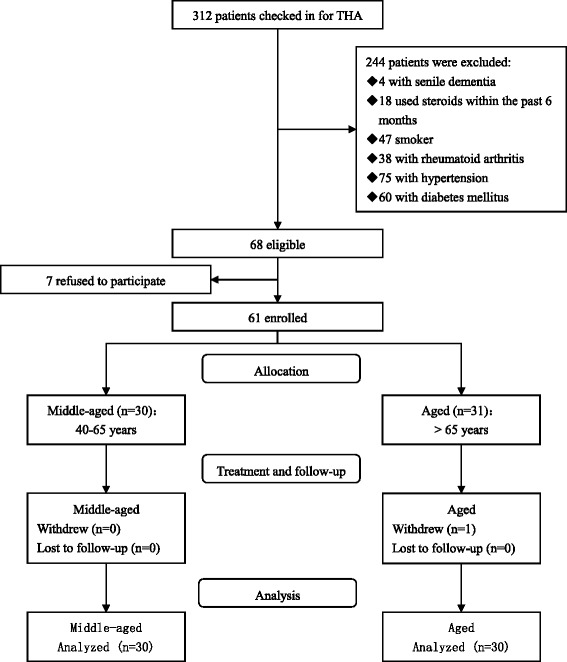
The chart of patient flow in the study

The patient screening and processing steps are illustrated in Fig. 1.

### Patient demographics and operative information

The two groups were well matched for gender, height, weight, BMI, and operating time (Table 1).

**Table 1:** Demographic and operative information

Parameters	Middle-aged	Aged	Z/χ^2^/*t*	*P*
Age(yrs)	53.93 ± 7.40	71.80 ± 5.85	Z = −6.658	<0.001
Gender(M/F)	11/19	13/17	χ^2^ = 2.411	0.121
Height(cm)	167.77 ± 11.63	163.30 ± 9.10	*t* = 1.657	0.103
Weight(kg)	66.33 ± 11.88	65.67 ± 11.70	*t* = 0.219	0.827
BMI	23.44 ± 2.46	24.44 ± 2.62	*t* = −1.525	0.133
Operative time(min)	64.87 ± 10.10	63.77 ± 11.33	*t* = 0.397	0.693

### Serial changes of cortisol

**Fig. 2:**
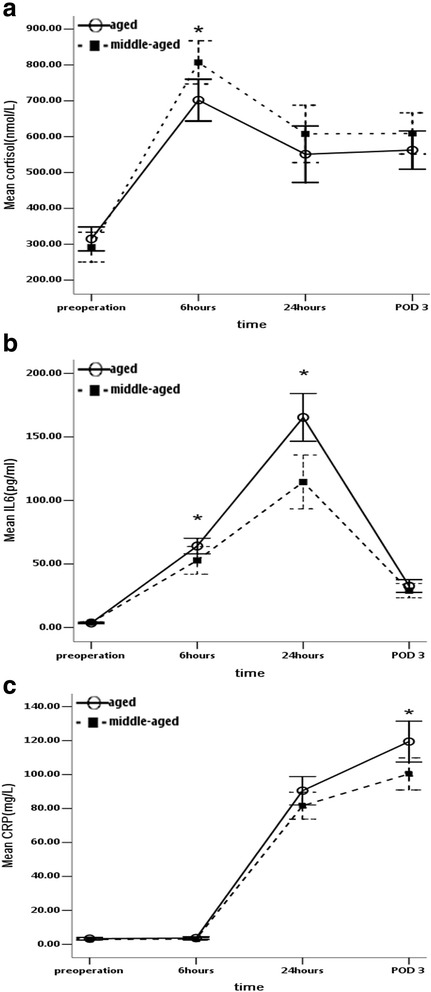
The stress response (cortisol, **a**) and inflammatory response (IL-6 and CRP, **b** and **c**) of middle-aged and aged patients preoperatively and at 6 h, 24 h and 3 days after THA. Asterisk (*) means the difference between the two groups reached statistical significance (*P* < 0.05)

The mean preoperative cortisol levels did not significantly differ between the two groups. Six hours after surgery, serum cortisol increased from 314.84 ± 89.65 nmol/L and 291.93 ± 111 nmol/L at baseline to 701.85 ± 156.32 nmol/L and 807.12 ± 161.34 nmol/L in the aged and middle-aged groups, respectively. Then, the cortisol concentrations dropped to 550.99 ± 210.73 nmol/L and 607.7 ± 214.31 nmol/L, respectively, at 24 h after the operation, which were still much higher than the baseline concentrations. The concentrations were almost the same at POD 3 (562.63 ± 142.7 nmol/L in the aged group and 609 ± 153.53 nmol/L in the middle-aged group) (Table 2). The preoperative serum cortisol concentrations and the cortisol levels at 24 and 72 h after surgery did not significantly differ between the two groups. However, serum cortisol was significantly lower six hours after the surgical procedure in the aged group than in the middle-aged group. Serum cortisol tended to be higher in the aged group preoperatively than in the middle-aged group; an opposite trend was seen postoperatively, although the differences were not significant except at 6 h after surgery (Fig. 2a).

**Table 2:** Cortisol, IL-6 and CRP levels before and 6 h, 24 h and 3 days after THA

Parameters		Aged	Middle-aged	t/Z	*P*
Cortisol (nmol/L)	Preoperation	314.84 ± 89.65	291.93 ± 111.00	−1.38	0.17
	6 h	701.85 ± 156.32	807.12 ± 161.34	2.57	0.01
	24 h	550.99 ± 210.73	607.70 ± 214.31	−0.72	0.47
	POD3	562.63 ± 142.70	609.00 ± 153.53	1.21	0.23
IL-6 (pg/ml)	Preoperation	3.54 ± 1.55	3.96 ± 1.59	1.05	0.30
	6 h	64.14 ± 16.43	52.81 ± 29.16	−2.48	0.01
	24 h	165.35 ± 50.50	114.53 ± 56.78	−3.47	<0.01
	POD3	32.60 ± 13.42	28.99 ± 15.01	−1.21	0.23
CRP (mg/L)	Preoperation	3.35 ± 1.88	2.87 ± 1.27	−0.61	0.54
	6 h	3.70 ± 2.27	3.14 ± 1.88	−0.98	0.33
	24 h	90.47 ± 22.44	81.56 ± 21.09	−1.59	0.12
	POD3	119.40 ± 32.06	100.27 ± 25.34	−2.56	0.01

### Serum IL-6 levels

The IL-6 concentration was 3.54 ± 1.55 pg/ml for the aged group and 3.96 ± 1.59 pg/ml for the middle-aged group before surgery, both of which were within the normal range. These concentrations increased to 64.14 ± 16.43 pg/ml and 52.81 ± 29.16 pg/ml, respectively, 6 h after surgery and continued to rise until they peaked at 24 h after surgery (165.35 ± 50.50 pg/ml and 114.53 ± 56.78 pg/ml). At POD 3, these concentrations plummeted to lower but still above-normal levels (32.60 ± 13.42 pg/ml and 28.99 ± 15.01 pg/ml, respectively) (Table 2). In contrast to the changes in serum cortisol, serum IL-6 was lower before surgery and higher at 6, 24, and 3 days after surgery in the aged group than in the middle-aged group, but significant differences between the two groups were observed only at 6 and 24 h (Fig. 2b).

### CRP concentration

The CRP levels were barely changed 6 h after THA compared with the preoperative measurements in both groups and fell within the normal range. The CRP concentration soared to 90.47 ± 22.44 mg/L (aged) and 81.56 ± 21.09 mg/L (middle-aged) 24 h after surgery and peaked at 119.40 ± 32.06 mg/L (aged) and 100.27 ± 25.34 mg/L (middle-aged) 3 days after the operation (Table 2). The aged group maintained higher blood CRP than the middle-aged group both before and after surgery, but the increments were subtle until 24 h after the operation in both groups. The difference became significant 3 days after surgery, at which time the blood CRP concentration was the highest within both groups. Additionally, the CRP concentration was virtually the same 6 h postoperatively and preoperatively (Fig. 2c).

### VAS evaluation

**Fig. 3:**
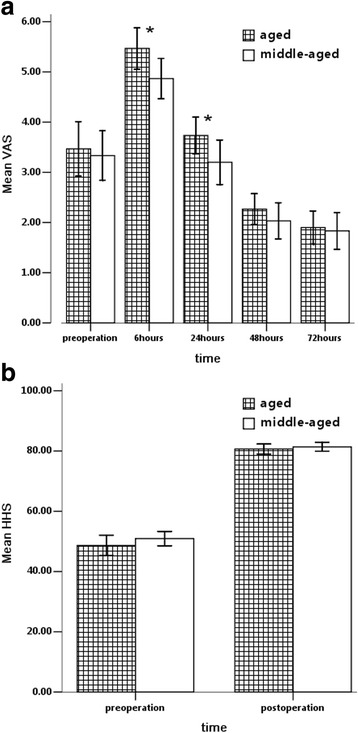
**a** The pain assessment (VAS) of middle-aged and aged patients preoperatively and at 6, 24, 48 and 72 h after THA. **b** The function assessment (HHS) of middle-aged and aged patients preoperatively and at 3 months after THA. *: *P* < 0.05 comparing the two age groups

The aged group scored 3.47 ± 1.46 in the preoperative VAS evaluation, whereas the middle-aged group scored 3.33 ± 1.32. Six hours after the operation, both groups experienced aggravated pain and scored 5.47 ± 1.11 and 4.87 ± 1.07, respectively. However, the scores in both groups decreased swiftly, with the aged group scoring slightly higher than their preoperative evaluation score (3.73 ± 0.98) and the middle-aged group scoring slightly lower than their preoperative rating (3.20 ± 1.19) 24 h after surgery. Then, the VAS scores in both groups fell to lower levels than their preoperative counterparts. The aged group felt more severe pain both before and after surgery than the middle-aged group, although significant differences were only shown at 6 and 24 h postoperatively, which was in accordance with the higher IL-6 levels in the aged group (Fig. 3a).

### HHS evaluation

The middle-aged and aged groups scored 50.93 ± 6.40 and 48.67 ± 9.11, respectively, before THA and then 81.37 ± 3.94 and 80.67 ± 4.44, respectively, after THA. Both groups showed evident improvement in the hip function assessment after THA, but no significant differences between the two groups were shown before or after THA (Fig. 3b).

### Possible correlations

**Fig. 4:**
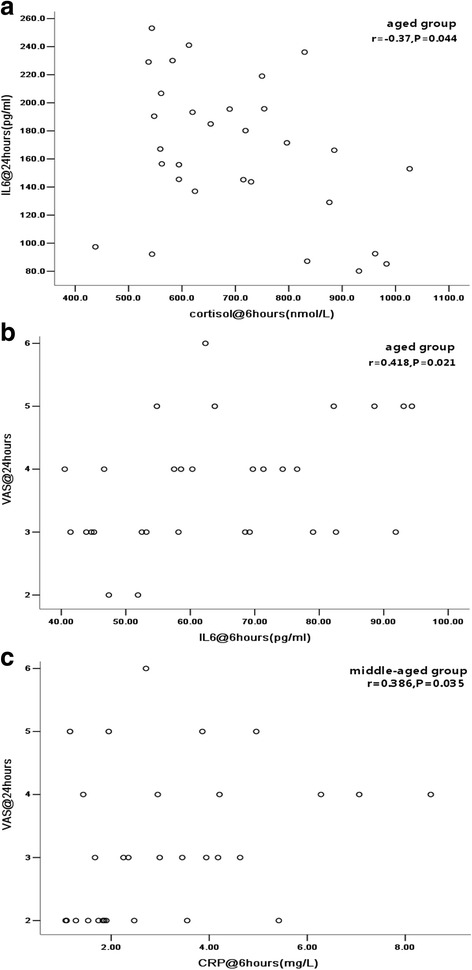
**a** Correlation between cortisol at 6 h after THA and IL-6 at 24 h after THA in the aged group. **b** Correlation between IL-6 at 6 h after THA and VAS at 24 h after THA in the aged group. **c** Correlation between CRP at 6 h after THA and VAS at 24 h after THA in the middle-aged group

The correlation analysis demonstrated only a possible weak correlation (*r* = −0.37, *P* = 0.04, Fig. 4a) between the cortisol concentration at 6 h and the IL-6 level at 24 h after THA in the aged group. A similar weak correlation (*r* = 0.42, *P* = 0.02, Fig. 4b) was detected between the IL-6 level at 6 h and the VAS score at 24 h after THA in the aged group. These correlations were not seen in the middle-aged group. Instead, the CRP concentration at 6 h after THA was weakly correlated with the VAS score at 24 h after THA in the middle-aged group (*r* = 0.39, *P* = 0.04, Fig. 4c). The rest of the data (including the cortisol, IL-6 and CRP levels; the VAS score at time points other than the aforementioned; and the HHS in both groups) were not correlated with each other.

## Discussion

To the best of our knowledge, no study in the major English language medical databases has reported differences in cortisol secretion and inflammatory responses between aged and middle-aged patients receiving THA. In this prospective observational study, we aimed to investigate the differences in the serum cortisol, IL-6 and CRP levels between aged and middle-aged patients.

This present study found that aged patients undergoing THA showed lower serum cortisol and higher IL-6 and CRP levels and more serious pain after surgery than the middle-aged patients. However, the differences between the two groups did not influence the HHS score 3 months postoperatively. The reasons for these results may be as follows. First, ageing is an inevitable physiological process that is accompanied by hypofunction of multiple body systems [13] and is related to musculoskeletal and arthritic disorders [20]. As a result, aged people often suffer suprachiasmatic nucleus impairment, which affects the hippocampus and weakens its ability to modulate the adrenocortical circadian rhythmicity [21] and stress response [22], possibly resulting in decreased circadian fluctuation of cortisol secretion [23] and a smaller increment of the serum cortisol concentration in aged people after THA. Second, IL-6 and CRP have been broadly used as markers of the inflammatory response after operations [24]. Because CRP and IL-6 are both related to the seriousness of tissue trauma, they are also recognized as biomarkers of the extent of surgery [25]. As a major orthopaedic surgery, THA will trigger significant increases in the serum IL-6 and CRP concentrations, which is consistent with the results of our study. The aged group exhibited a stronger inflammatory response, suggesting that aged patients experienced a greater stress response. Hall GM et al. [26] reported that a lower IL-6 concentration more rapidly and exactly predicted the ability to walk 10 m and 25 m, whereas a lower CRP concentration was correlated with less pain at the time of discharge. Moreover, regardless of the nature of pain, all pain originates from inflammatory responses [27]. Thus, it is beneficial for the rehabilitation of aged patients to have a weaker inflammatory response. Third, the postoperative functional outcome of patients undergoing THA is affected by multiple factors and not only by endogenous or exogenous corticosteroids.

IL-6 and CRP are makers of inflammation, and inflammation triggers pain. Cortisol has anti-inflammatory effects; therefore, higher cortisol levels should suggest stronger anti-inflammatory efficacy and be directly correlated with lower IL-6 and CRP levels and indirectly correlated with the VAS score, at least in theory. However, in our study, only very weak correlations were found. These weak correlations could be coincidental correlations in our data. The small sample size may partially account for this unsatisfying result. There were several outliers in our data that could have weakened our results. Moreover, the use of NSAIDS for pain management could have inhibited the inflammatory response to a certain extent, thereby rendering the correlation weak or even non-significant.

Due to their potent effects on the inflammatory and immune responses, glucocorticoids have been broadly used to relieve the stress response caused by surgery [28]. In a prospective study comparing 40 patients undergoing elective total knee arthroplasty for OA, injection of a small dose of dexamethasone into the peri-articular soft tissues significantly decreased the CRP concentration on the third postoperative day and IL-6 in the drainage fluid 24 h after the operation [29]. Peter et al. [30] found that the perioperative administration of corticosteroids significantly decreased serum IL-6 at 6 and 24 h after surgery. Additionally, glucocorticoids improved postoperative nausea and pain and shortened the length of hospitalization [29, 31]. In our study, the aged patients showed lower endogenous cortisol after surgery but suffered more severe pain at 6 h postoperatively, implying that endogenous cortisol could not relieve the stress response and that exogenous corticosteroid should be used to improve the postoperative outcome. Unfortunately, corticosteroid is also associated with adverse events, such as hyperglycaemia, poor wound healing, deep infection and delayed bone healing [32–36]. A meta-analysis by Toner AJ et al. confirmed that perioperative administration of glucocorticoids increased the blood glucose concentration [33]. In addition, even a single surge of perioperative blood glucose is directly related to infectious and non-infectious complications [34]. In animal models, glucocorticoids significantly impaired bone healing and reduced bone turnover [35, 36]. Although whether the perioperative use of corticosteroid produces adverse effects, such as impaired wound healing and deep infection, is controversial, some studies have suggested that low-dose and short-term administrations of corticosteroid should lessen these concerns [29–31, 37–40]. Glucocorticoid can relieve the stress response and alleviate postoperative pain but does not improve the functional outcome [38]. In the present study, there was no differences in the HHS between the two groups, implying that the postoperative functional outcome of patients undergoing THA was affected by multi-factors and not only by endogenous or exogenous corticosteroid.

Several limitations should be mentioned. The number of patients recruited in our study was small, and larger samples may be needed to reveal differences between some of the variables. Celecoxib and parecoxib were administered to the patients, which suppressed the inflammatory response and affected the serum IL-6 and CRP levels. The HHS may not have been sensitive enough to reflect minute differences in the surgical outcome and patient satisfaction between the two groups. Two other limitations of our study are the short observational time and the lack of standardized follow-up methodology. To confirm and validate the results of our study, studies with a large sample size and multi-centre randomized controlled trials are needed.

## Conclusion

Aged patients undergoing THA exhibited a lower level of endogenous cortisol production at 6 h postoperatively and a more severe inflammatory response (IL-6 at 6 and 24 h and CRP at 3 days after surgery) and postoperative pain (VAS at 6 and 24 h after surgery). Further studies of perioperative glucocorticoid usage in aged patients should be conducted to demonstrate the benefits and possible side effects.

## Abbreviations

CRP: C-reactive protein
HHS: Harris hip score
IL-6: interleukin-6.
NSAIDs: Non-steroidal anti-inflammatory drugs
OA: osteoarthritis
POD: Postoperative day
THA: Total hip arthroplasty
VAS: Visual analog scale
